# Histoarchitecture of the fibrillary matrix of human fetal posterior tibial tendons

**DOI:** 10.1038/s41598-022-19695-3

**Published:** 2022-10-26

**Authors:** Rodrigo Sousa Macedo, Walcy Rosolia Teodoro, Vera Luiza Capellozzi, Dov Lagus Rosemberg, Rafael Barban Sposeto, Cesar de Cesar Netto, Jonathan T. Deland, Nicola Maffulli, Scott J. Ellis, Alexandre Leme Godoy-Santos

**Affiliations:** 1 Lab. Prof. Manlio Mario Marco Napoli, Departamento de Ortopedia e Traumatologia, Hospital das Clinicas, Faculdade de Medicina, Universidade de São Paulo, R. Dr. Ovídio Pires de Campos, 333-Cerqueira César, São Paulo, SP 05403-010 Brazil; 2 Rheumatology Division of the Hospital das Clinicas, Faculdade de Medicina, Universidade de São Paulo, FMUSP, São Paulo, Brazil; 3 Department of Pathology of the Hospital das Clinicas, Faculdade de Medicina, Universidade de São Paulo, FMUSP, São Paulo, Brazil; 4 Foot and Ankle Services, Department of Orthopedics and Rehabilitation, University of Iowa, Carver College of Medicine, John Pappajohn Pavilion (JPP), Room 01066, Lower Level, 200 Hawkins Drive, Iowa City, IA 52242 USA; 5 Department of Foot and Ankle Surgery, Hospital for Special Surgery, New York, NY USA; 6 Department of Medicine, Surgery and Dentistry, University of Salerno, Via S. Allende, 84081 Baronissi, SA Italy; 7 Barts and the London School of Medicine and Dentistry, Centre for Sports and Exercise Medicine, Mile End Hospital, Queen Mary University of London, 275 Bancroft Road, London, E1 4DG UK; 8 School of Pharmacy and Bioengineering, Keele University School of Medicine, Stoke on Trent, ST5 5BG UK

**Keywords:** Cells, Musculoskeletal system, Musculoskeletal system

## Abstract

Adult tendons are highly differentiated. In mature individuals, tendon healing after an injury occurs through fibrotic tissue formation. Understanding the intrinsic reparative properties of fetal tendons would help to understand the maturation tissue process and tendon tissue repair. The present study evaluated the evolution of histoarchitecture, cellularity and the distribution of collagens I, III and V in the posterior tibial tendon in human fetuses at different gestational ages. Morphological profiles were assessed in nine fresh spontaneously aborted fetuses (Group I: five fetuses aged between 22 and 28 weeks of gestation; Group II: four fetuses aged between 32 and 38 weeks of gestation), characterized by a combination of histology, fluorescence and immunohistochemistry. In Group I, the posterior tibial tendon showed statistically significant greater cellularity and presence of collagen III and V than in Group II tendon, which showed a predominance of collagenous I and a better organization of the extracellular matrix compared with Group I tendons. In addition, a statistically significant higher rate of CD90, a marker of mesenchymal cells, was found in Group I tendons. In fetuses with gestational age between 22 and 28 weeks, the posterior tibialis tendons showed a thin and disorganized fibrillar structure, with an increase in collagen III and V fibers and mesenchymal cells. In the posterior tibialis tendons of fetuses with gestational age between 32 and 38 weeks, the fibrillar structure was thicker with a statistically significant increase in type I collagen and decreased cellularity.

## Introduction

The posterior tibial tendon (PTT) is the main inverter of the ankle and foot and the primary dynamic stabilizer of the medial plantar arch, acting in conjunction with the capsulo-ligamentous passive stabilizers^1^. Posterior tibial tendinopathy is very common, especially in women, in part due to hormonal changes related to the postmenopausal period, with a prevalence of 10% in the seventh decade of life, restricting activity and affecting the quality of life of this population^1–4^. New factors have been implicated in the genesis of pathology, with accelerated development of new techniques, implants, and rehabilitation protocols; however, the posterior tibial tendon remains the primary dynamic arch stabilizer, and tissue healing remains a fundamental factor to achieve optimal clinical results^5^.

Adult tendons are highly differentiated structures that, after an injury, react through a reparative process that results in the formation of a fibrotic scar due to its low regeneration capacity^6,7^. Tissue alterations are represented by degenerative tendinosis characterized by: neovascularization, mucin deposition and an increase in the quantity and activity of fibroblasts, reflected respectively by the increase in cellularity, proline and hydroxyproline in the degenerated tendon. Another relevant alteration is the modification of the composition of the different types of collagens in the tendon tissue, with the reduction of type I collagen and a proportional increase of types III and V. These modifications lead to the breakdown of the linear formation of collagens fibers, reflecting a less elastic and resistant tissue^8–12^.

In contrast, fetal tendon responses to injury are more efficient. It is well established that fetal tissue, especially in the early and middle stages of pregnancy, responds to injury differently from adult tissue^13,14^. In general, fetal wound healing occurs at a faster rate and in the absence of scar formation. This observation was confirmed in animal models and different tissues, including the tendon^15^. Intrinsic fetal regenerative properties are shown by data on matrix composition and changes in tenocyte synthetic activity, which are directly correlated with tissue maturation and aging^16–18^.

During fetal development, tendons undergo significant changes that transform a plastic tissue into a highly differentiated structure. Tendon maturation is characterized in some studies by its morphological and histoarchitectural characteristics, which include cellularity and the organization and distribution of collagens, as well as evaluating the gene expression of factors that correlate with fetal maturity such as Scleraxis B and Tenomodulin. In general, during development, these genes suffer a reduction in expression, which would justify the lower tissue regenerative capacity after maturation^19,20^. However, most studies on tendon organization and function analyze the tissue of mice, rats, rabbits, horses, and sheep, with a limited number of studies on human fetal tendon histomorphology^19,21–23^.

We hypothesized that there is a difference in the composition and arrangement of the extracellular matrix of the posterior tibial tendon of human fetuses at earlier stages when compared to the lateral gestational ages. The present study evaluated the evolution of histoarchitecture, cellularity, mesenchymal stem cell markers, and the distribution of collagens I, III and V in the posterior tibial tendon in human fetuses of different gestational ages.

## Methods

Our institution's scientific and research ethics committees approved all the procedures described in the present study (Scientific Committee of the Institute of Orthopedics and Traumatology, Faculty of Medicine, University of São Paulo under protocol number 1340 and consubstantial opinion of the Ethics and Research Committee under number 3.031.872). Signed parental informed consent was obtained to examine fetuses obtained from spontaneous abortions and stillbirths. All experiments were performed in accordance with relevant guidelines and regulations, including the Declaration of Helsinki. Gestational ages were determined by the head, buttock length, foot length and fetal weight, and the gestational age mentioned by the parents when signing the consent form (ICF)^24^.

### Inclusion criteria

Spontaneously aborted fetuses with a gestational age between 22 and 38 weeks.

### Non-inclusion criteria

Fetuses less than 22 weeks old, given the difficulties in the dissection of the early specimens and the higher chance of malformations or anomalies evident on external inspection or after autopsy.

We obtained a total of 9 fetuses divided into two groups, Group 1: 5 fetuses—22–28 weeks of gestation (mean 24.80 weeks gestational age) and Group 2: 4 fetuses—32–38 weeks of gestation (mean 33.75 weeks of gestational age).

The PTT was collected from both sides of each fetus. These specimens underwent histoarchitecture and histomorphometric analysis of the extracellular matrix components, including collagens I, III and V.

### Dissection and specimen preparation

**Figure 1:**
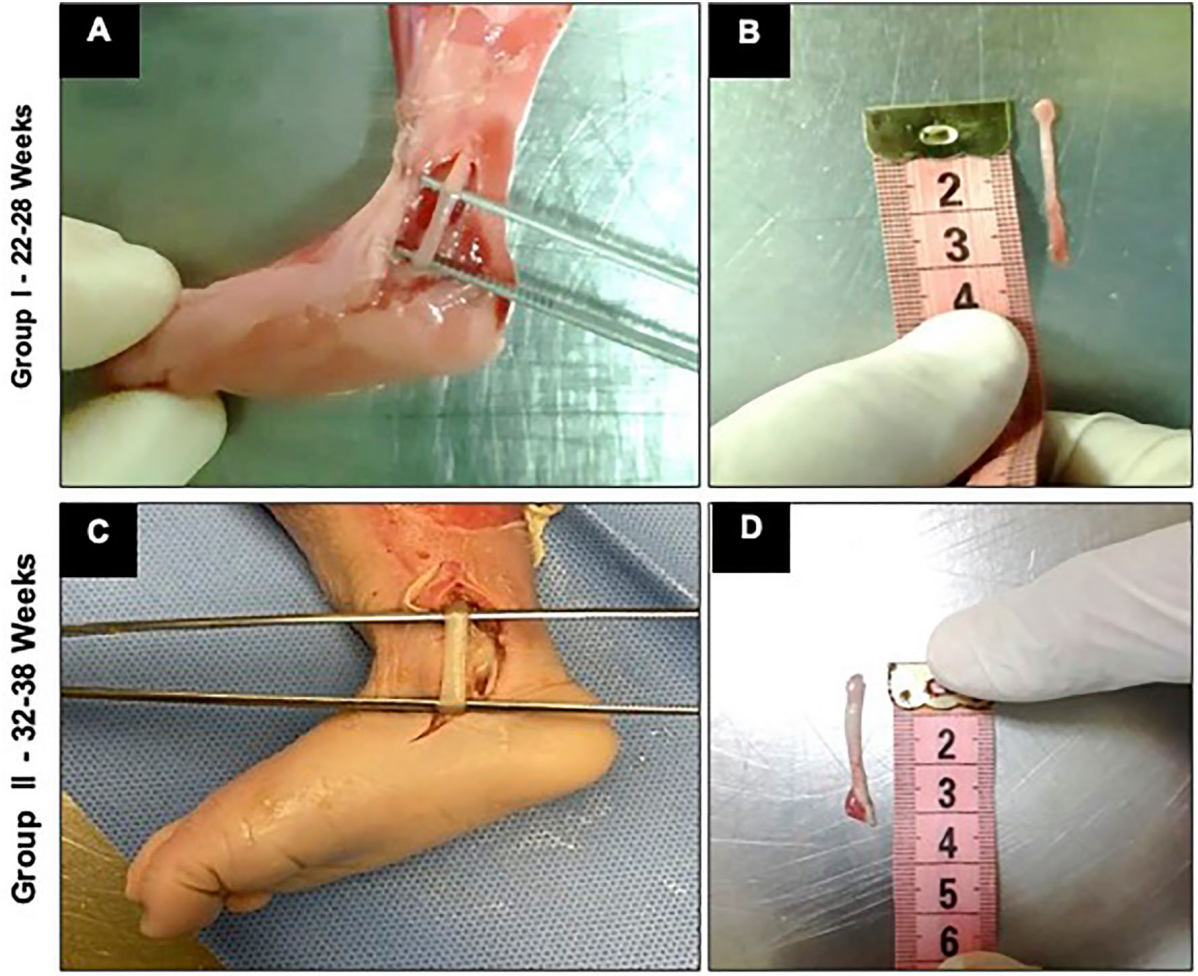
Dissection of tendon specimens. (**A**,**B**) Fetuses of Group I from 22 to 28 weeks; (**C**,**D**) Fetuses of Group II from 32 to 38 weeks. (**A**,**C**) Dissection of the fetus ankle of the tibialis posterior tendon from its origin to its insertion. (**B**,**D**) Measurement of the sample obtained from one of the lower limbs of a fetus.

We performed the dissections and resections of the tendons of human fetuses' fragments at the Death Verification Service of the Capital of the University of São Paulo. With the aid of a 15 mm curved scalpel blade, we made an incision of approximately 1 cm over the TTP in the posteromedial region of the ankle. The posterior tibial tendon was identified and was harvested from its origin to insertion (Fig. 1). For the purposes of the histological and morphometric analyses, human fetal tendon samples were immersed in buffered formalin 10% with a Sodium Phosphate buffer solution at pH = 7.0 for staining with hematoxylin–eosin (H&E) and Picrossírius. The solution consists of Sodium Chloride (Code S9888), Potassium Chloride (Code P3911), Potassium Phosphate Monobasic (Code P5655), and Dibasic Sodium Phosphate (Code P71645).

### Optical microscopy

Tendon sections (3–4 μm) were used in the deparaffinization process in ethanol and hydrated in graded ethanol. Subsequently, they were stained with hematoxylin guidance (H&E) and instrument evaluations and evaluations at Bx51, Tokyo, Japan) for B51 structure and tissue cell age. Subsequently, from an analysis of tissue collagen in optical analysis, samples were stained by Picrosirius, which is a selective connective tissue staining that allows a qualitative analysis of the collagen fibers of connective tissue, prepared from Sirius red 0.2% here epicry acid solution (Direct Red 80, CI375, Milwaukee, WI).

### Collagen types immunofluorescence

For immunostaining of collagen types I, III and V, 3–4 µm sections of tendon tissue samples were adhered to slides with aminosilane (Sigma Chemical Co.; St. Louis, Missouri, USA). They were immersed in xylene and rehydrated in decreasing concentrations of ethanol. The immunogenic sites were exposed to enzymatic treatment with porcine gastric mucosa pepsin (P7000; Sigma Chemical Co.; St. Louis, Missouri, USA) at 10 mg/ml concentration in 0.5 N acetic acid, pH 2.2 for 45 min, at 37 °C. After successive washes with PBS, the slides were incubated in 5% bovine albumin (BSA) and diluted in phosphate buffer pH 7.0 for 30 min. They were subsequently incubated overnight at 4 °C with polyclonal rabbit anti-human Collagen I (1:100, Rockland, Carlsbad, CA, USA), anti-human Collagen III (1:200, Rockland, Carlsbad, CA, USA)), and anti-human Collagen V antibodies (1:2000, Rockland, Carlsbad, CA, USA) diluted in BSA (bovine albumin, Sigma Chemical Co., St. Louis, MO, USA) and stained with goat antibody ALEXA FLUOR 488 anti-rabbit IgG (Invitrogen, Carlsbad, CA, USA) diluted 1:200 in a PBS solution containing 0.006% Evans blue. As a negative control, the primary antibody was replaced by PBS. After washing with PBS/0.05% Tween20, the slides were again incubated with 1 µg/ml Hoechst 33258 bisbenzimide (DAPI) (Invitrogen, Carlsbad, CA, USA) to evidence the cell nucleus. Finally, slides were mounted with a glycine buffer in PBS (v/v) and analyzed under a fluorescence microscope (Olympus BX51, Olympus Co, St Laurent, Quebec, Canada)^12,25^.

### Mesenchymal stem cell immunostaining

To analyze mesenchymal stem cells from the tendon tissue, 3–4 µm sections were adhered to slides with aminosilane (Sigma Chemical Co.; St. Louis, Missouri, USA). The slides were immersed in xylene and rehydrated in decreasing concentrations of ethanol. Subsequently, a 0.3% hydrogen peroxide solution was used four times for 5 min to inhibit the activity of endogenous peroxidase, and the antigenic recovery was immediately processed. The immunogenic sites were exposed to enzymatic treatment with porcine gastric mucosa pepsin (P7000; Sigma Chemical Co.; St. Louis, Missouri, USA) at a concentration of 0.4% in glycine buffer pH 2.2 for 30 min at 37 °C, and then incubated with CD90 monoclonal primary antibody (Abcam Ab 92574) diluted 1:200 in 0.01% BSA, overnight at 4 °C. According to the manufacturer's recommendations, the reaction was developed using a biotin–streptavidin–peroxidase (Novolink Polymer Detection Systems Kit, Leica Biosystems, UK). After that, 3,3 diaminobenzidine (Sigma Chemical, St Louis, MO) was used as the chromogen and counterstained with Harris hematoxylin (Merck, Darmstadt, HE Germany). The IgG isotype was used as a negative control. To access uniform and proportional tendon samples, ten fields were randomly analyzed in the tendon at ×1000 magnification for CD90 expression, and the cells count was performed by a manual point count in each field system software Image Pro-Plus 6.0), composed of an Olympus camera (Olympus Co, St Laurent, Quebec, Canada) coupled to an Olympus microscope (Olympus BX51), from which the images were sent to an LG monitor utilizing a digitizing system (Oculus TCX, Coreco, Inc., St. Laurent, Quebec, Canada) The results are reported as the percentage of positive cells in the tendon per micrometer square^26^.

### Histomorphometry of tendons

Photomicrographs at magnification 400× were obtained from ten non-overlapping fields of view per section under a fluorescence microscope (Olympus BX51, Olympus Co, St Laurent, Quebec, Canada). The images were processed through Image Pro-Plus 6.0 software (NCH Software Inc., Greenwood Village CO, USA), and the immunofluorescence density of collagen I, III, and V fibers were measured. The threshold for identification of collagen fibers was given for all slides after the contrast was increased to the point where the fibers were clearly identified as green bands. Its density was expressed as the ratio between the measurement of the fibers by the total area studied 100×. The microscopic fields of the slides were quantified, and the results were shown as the average of these fields.

### Statistical analysis

Data are presented as the mean ± standard deviation of the mean. Statistical analysis was performed using *GraphPad Prism 6.0 software* (GraphPad Prism, Inc., San Diego, CA, USA), and P-values < 0.05 were considered significant. Statistical differences between groups were determined by the *Mann–Whitney U* test for an area of the fraction occupied by mesenchymal cells and immunofluorescence for collagen I, III and V of the tendons. *Post-hoc* tests, corrected for multiple comparisons with the Tukey-Kraemer fit, were used to locate significant differences when significant main effects or interaction effects were shown. *Spearman* correlation was performed between cells and collagen types.

## Results

### Cell distribution in human fetal tendons

**Figure 2:**
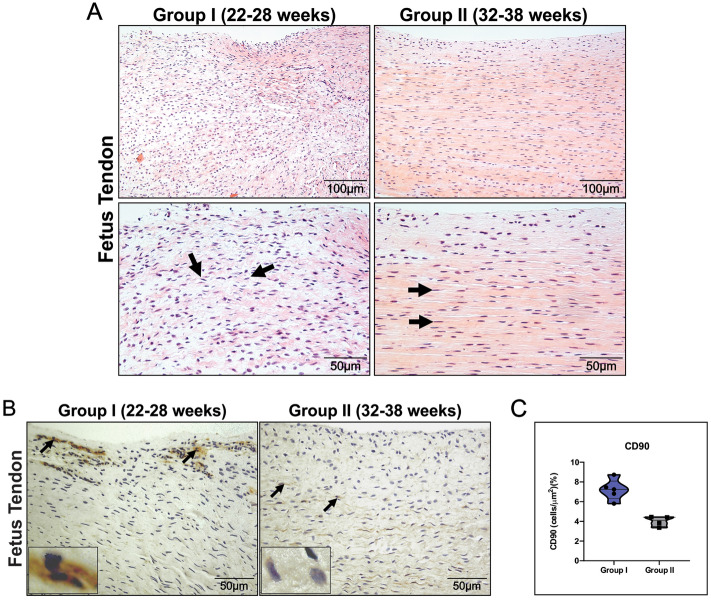
Cell distribution in human fetal tendons. (**A**) Cell distribution in human fetal tendons—Human fetal tendon samples from Group I (22–28 weeks; n = 5) and Group II (32–38 weeks; n = 4) show typical histoarchitecture appearance in H&E preparations. At lower magnification, a large number of cells immersed in the fibrillar matrix can be seen in Group I in relation to the tendons in Group II. At higher magnification, note the cells distributed among the collagen fibers in the tendons of Group I (arrows) compared to the tendons of Group II (arrows) (insert) arranged in a linear orientation along the collagen bundles. (**B**) Immunostaining of CD90 in Group I compared to human fetal tendons in Group II. Note a high expression of CD90 positive cells throughout the tendon matrix in human fetal tendons in Group I (arrows) (insert) and in (**C**) Graphic representation of CD90 measurement (mean + standard deviation), showing a significant increase in the number of positive cells in Group I compared to Group II (7.20 ± 1,06 *vs.* 4.01 ± 0.51 cell/μm^2^; p < 0.015). This graph shows the mean and the value found in the analysis of the dispersion of each specimen in the sample. *GraphPad Prism 6.0 software: Mann Whitney U test; P-values* < *0.05 were considered significant.*

Group I (22–28 weeks) had tendons with a large number of cells with an asymmetric distribution, mainly in the endotenon region, around the tendinous tissue and immersed in collagen bundles, following a pattern of thick scaffolding. In Group II (32–38 weeks), tendons with a lower degree of cellularity were found, with increased parallel or linear orientation of collagen bundles (Fig. 2A). Regarding the presence of mesenchymal cells, we found an intense expression of CD90 in human tendons in Group I compared to immunostaining in Group II. A high expression of CD90-positive cells in the tendon tissue is characterized by intense cytoplasmic immunostaining in these cells (Fig. 2B). Furthermore, the histomorphometric results showed more CD90 in human fetal tendons in Group I compared to Group II (7.20 ± 1.06 vs. 4.01 ± 0.51; P < 0.0159), this marker was found mainly in the endotenon (Fig. 2C).

### Collagen molecular architecture and cells amount depends on gestational fetus age

**Figure 3:**
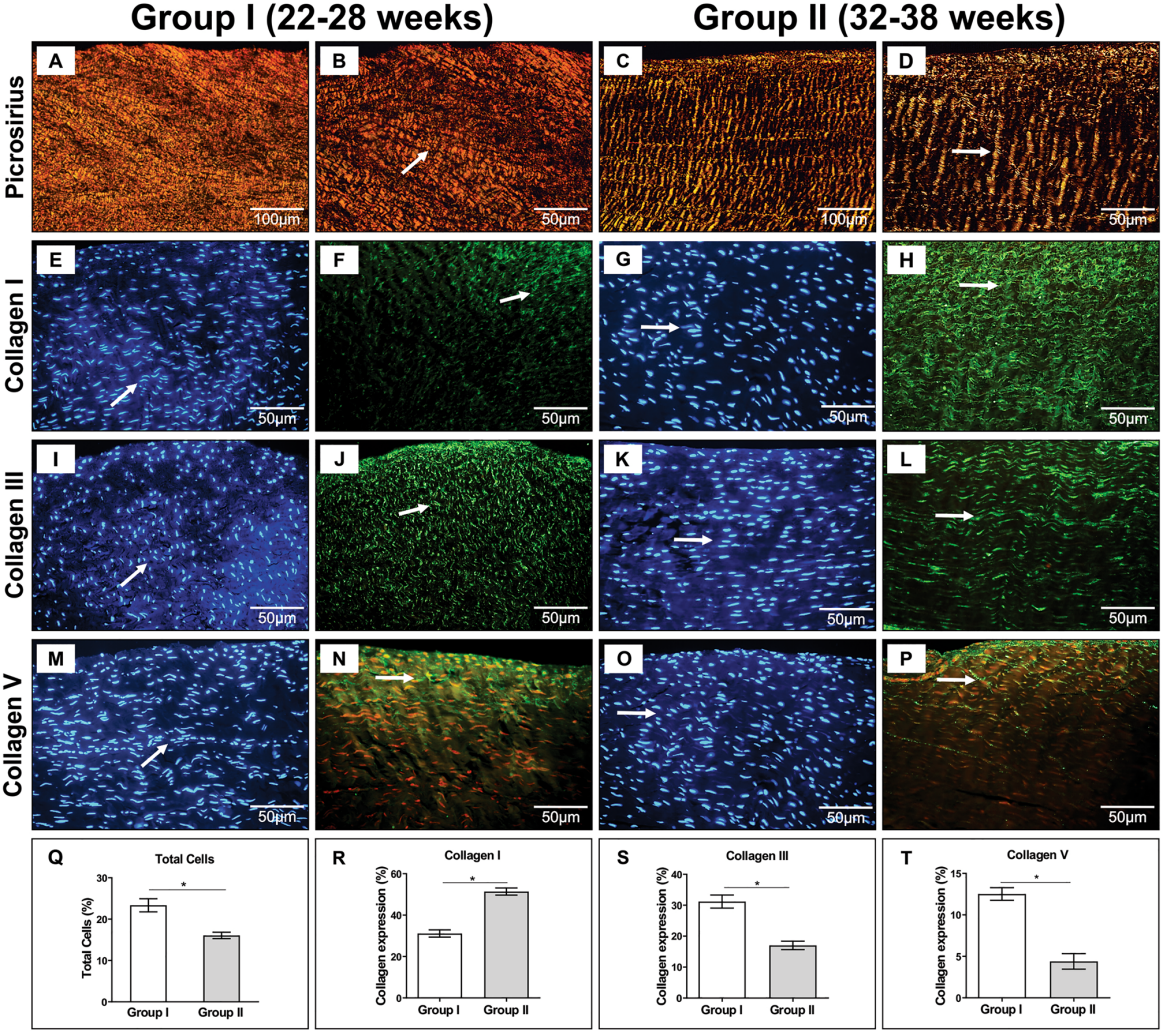
Collagen molecular architecture and cells distribution. (**A**,**B**) note reddish-orange birefringent collagen fibers analyzed by picrosirius under polarization in Group I (n = 5) human fetal tendons with a fragmented and disarranged pattern. In contrast, the collagen fibers stay in parallel or linear orientation in Group II (n = 4) tendons, (**C**,**D**) (arrows). (**E**,**I**,**M**) fluorescence microscopy by DAPI staining to identify the cell nucleus, with a large amount of cells with diffuse pattern in Group I tendons and in (**G**,**K**,**O**) demonstrate Group II tendon specimens with uniform and linear arrangement of the nuclei (arrows). (**F**,**J**,**N**) Immunostaining to collagen I, III and V visualized under fluorescence microscopy of Group I, demonstrating the reticulated pattern of collagen types. In contrast, (**H**,**L**,**P**) uniform green fluorescence and parallel orientation of collagen type I, III and V fibers in all areas of Group II tendons. (**Q**) A graphic representation of the total number of cells in Group I compared to Group II tendons (23.35 ± 1.74 vs. 16.07 ± 0.77; p < 0.04). A graphic representation of the amount (percentage of total tendon area, mean and standard deviation) of collagen I (51.40 ± 1.71 vs. 31.11 ± 1.74; p < 0.016) (**R**), collagen III (31.19 ± 2.10 vs. 17.02 ± 1.37; p < 0.015) (**S**) and collagen V (12.52 ± 0.76 vs. 4.392 ± 0.76; p < 0.016) (**T**) in the Group I in compared to Group II tendons. GraphPad Prism 6.0 software: Mann Whitney U test; P-values < 0.05 were considered significant.

In Group I, a network of reddish-orange birefringent fibers in Picrosirius staining was identified, with a fragmented and broken pattern covering the entire surface of the tendon (Fig. 3A,B). In contrast, the collagen fibers in group II human fetal tendons showed a parallel or linear orientation of the tissue collagen bundle (Fig. 3C,D).

Human fetal tendons in Group I showed greater cellularity, with a disordered pattern in all tendon tissues (Fig. 3E,I,M). In Group II tendons, the number of cells decreased, and a linear and uniform pattern oriented in parallel, following the tissue-oriented fibrillar matrix, was evident (Fig. 3G,K,O).

Through histomorphometric analysis, there was a significant increase in cell content in human fetal tendons in Group I compared to tendons in Group II (23.35 ± 1.74 vs. 16.07 ± 0.77; p < 0.0357) (Fig. 3Q). The immunofluorescence analysis of collagen I showed green fluorescence for collagen type I, with diffuse fiber distribution in all areas of the tendon for Group I. On the other hand, tendons in Group II exhibited marked green fluorescence for collagen type I, with parallel or linear orientation of the collagen bundles (Fig. 3F,H). Quantitative results showed a significant increase in collagen I in Group II tendons compared to Group I tendons, respectively (51.24 ± 1.71 vs. 31.11 ± 1.74; p < 0.0159) (Fig. 3R). In addition, immunofluorescence analysis of collagen types III and V showed a finely reticulated network of collagen fibers in human fetal tendons in Group I, with diffuse fiber distribution in all tissue areas (Fig. 3J,N). In contrast, Group II tendon specimens showed prominent fluorescence for collagen types III and V arranged in a fine and regular fibrillar fashion with better organization of the extracellular matrix (Fig. 3L,P). There was a significantly greater amount of type III collagen in the tendons of Group I compared to group II, respectively (31.19 ± 2.10 vs. 17.02 ± 1.37; p < 0.015), as well as type V collagen (12.52 ± 0.76 vs. 4.39 ± 0.76; p < 0.016) (Fig. 3S,T).

## Discussion

As in other connective tissues, the human posterior tibial tendon undergoes morphological and molecular changes during its development, which involve both its cells and the extracellular matrix. The process of in utero maturation of the posterior tibial tendon is accompanied by a progressive decrease in cellularity and modification of tissue structure. We observed a significantly greater number of tenocytes per field in the immature tendon, confirming the findings of studies in equine and sheep tendons^16,19^. This is the first study to perform this evaluation in the posterior tibial tendon of human fetuses.

During the process of fetal development, there was a reduction in the cellularity of the posterior tibial tendon that accompanied tendon maturation. The endotenon was more robust in younger fetal tendons compared to more mature ones, and cell characteristics also changed as the tendon matured. In fact, the specimens from Group I presented heterogeneous cell shapes and in greater quantity when compared to those from Group II, which presented cells with a more fusiform shape and in smaller numbers. Avilion et al. and Russo et al. have described similar findings in animal tendon specimens^27,28^. The fact that there is no evidence of pyknosis or nuclear fragmentation under microscopy makes us suppose that this reduction is unrelated to apoptosis. Hosaka et al. and Chuen et al. present similar results in horse digital flexor and human patellar tendons, respectively^17,18^.

In our study, the posterior tibial tendon of fetuses at different gestational ages presented collagen types I, III and V in different amounts and a distinct distribution pattern. Tresoldi et al. state that fibrillogenesis begins during embryogenesis and continues after birth with the assembly of type I collagen molecules, which follow linear and lateral growth associated with collagen interactions with proteins such as other collagens and proteoglycans^10^. Initially, collagen molecules assemble to form immature fibril intermediates, and after this molecular assembly, fibril intermediates assemble end-to-end to form longer fibrils, consistent with mechanically functional mature fibrils. These data corroborate findings in the posterior tibial tendon of human fetuses, in which the amount and pattern of organization of type I collagen increased in more mature fetuses compared to younger ones.

Younger fetuses had collagen type III in greater amounts compared to more mature fetuses. Tozer and Duprez state that collagen III expression gradually decreases during development and that its high expression in the early stages suggests a role in the initial assembly of the fibril. Furthermore, its expression is elevated after tissue injury, suggesting that this collagen may play a role in the healing process, perhaps by stimulating fibrillogenesis^29^.

Romanic et al. evaluated that collagen III, in the form of procollagen III, can regulate the diameter of collagen I fibrils, coating their surface, thus allowing longitudinal growth, but not lateral growth of fibrils with eventual thickening of the tissue tendon^30^.

Hansen et al. demonstrate that type V collagen plays the role of a molecular ligand between collagen I fibrils or between fibrils and macromolecules depending on their respective distribution in different tissues^31^. Thus, it is associated with the quantity and quality of the distribution of collagen I fibers and, consequently, of the tendon. Connizzo et al. demonstrated that, after the deletion of the genes responsible for the production of type V collagen in mice, an assembly of fibrils of large diameter and wide distribution occurs, characteristics similar to the fibrils produced in connective tissues with low concentrations of this collagen. This suggests that type V collagen levels regulate fibril diameter and that its reduction may be sufficient to alter fibril assembly so that abnormally large diameter fibrils are deposited in the matrix^32^. In our study, younger fetuses had a higher amount of type V collagen when compared to more mature fetuses. This corroborates the data presented in the literature and suggests a greater ability to regulate and organize immature tissue.

Younger fetal tendon tissue samples showed a greater amount of cells immunostained for CD90, one of the markers of mesenchymal stem cells, compared to more mature tendons^33^. The presence of these cells was observed mainly close to the blood vessels, as demonstrated by Lui et al. in their evaluation of adult tendons, suggesting that they may respond to local and systemic regulatory signals^34^. Russo et al. also demonstrated a high expression of markers that characterize mesenchymal cells in fetal tendons of younger sheep when compared to more mature and adult fetal tendons^19^. Adult mesenchymal stem cells are able to differentiate into: bone, cartilage, muscle, medullary stroma, tendon, ligament, fat, and other connective tissues in a sequence of lineage transitions. Caplan et al. demonstrate that bioactive molecules secreted by these cells were able to promote neovascularization, migration, immunoregulation, cell proliferation, synthesis, and remodeling of the extracellular matrix in the tissue^35^. However, he states that it is still unclear whether tendon stem cells work to replace damaged tendons or to establish a microenvironment for injury repair. Still, both can occur in vivo after tendon injury.

In the present study, the characterization of mesenchymal stem cells aimed to identify whether the constitution of the fibrillar matrix of the tendon could be linked to the increase of these cells during the development of the tendon tissue of the human fetus. However, other markers that characterize this cell lineage would be necessary to support this hypothesis. On the other hand, the indication of a greater amount of cells that express CD90 may be a strong indication of the participation of these cells in the maturation of the tendon of the human fetus.

Goldman et al. and Russo et al. have already demonstrated a greater association of the amount of TGFβ with the production of collagen I and a greater association with mesenchymal stem cells in the endotenon^19,36^. Our attempt at immunostaining TGFβ in a human tendon by immunohistochemistry showed a nonspecific and difficult to interpret the result in tissue. We believe that the fact that the fetuses were removed from the death verification service may have had some influence on this tissue.

A deeper understanding of the intrinsic properties of the response of fetal tissue to injury may allow for the modulation of the response of mature tendon tissue to injury. There are undoubtedly many more molecules involved in this response than can be examined in a single investigation, which is a weakness of the present work. Nevertheless, our study begins to provide information on the complex mechanisms that control the process of maturation and organization of human tendons, and the information from this project can be the starting point for the development of innovative therapies to minimize the formation of scars after tendon injury in humans.

## Supplementary Information

Supplementary Information.

## Data Availability

All data generated or analysed during this study are included in this published article [and its supplementary information files].

## References

[1] Bloome DM, Marymont JV, Varner KE (2003). Variations on the insertion of the posterior tibialis tendon: A cadaveric study. Foot Ankle Int..

[2] Kohls-Gatzoulis J, Woods B, Angel JC, Singh D (2009). The prevalence of symptomatic posterior tibialis tendon dysfunction in women over the age of 40 in England. Foot Ankle Surg..

[3] Ross MH, Smith M, Plinsinga ML, Vicenzino B (2018). Self-reported social and activity restrictions accompany local impairments in posterior tibial tendon dysfunction: A systematic review. J. Foot Ankle Res..

[4] Pontin PA, Nogara PRB, Fonseca FCP, Cesar Netto C, Carvalho KC, Soares Junior JM (2018). ERα PvuII and XbaI polymorphisms in postmenopausal women with posterior tibial tendon dysfunction: A case control study. J. Orthop. Surg. Res..

[5] Myerson MS, Thordarson DB, Johnson JE, Hintermann B, Sangeorzan BJ, Deland JT (2020). Classification and nomenclature: Progressive collapsing foot deformity. Foot Ankle Int..

[6] Lin TWTW, Cardenas L, Soslowsky LJLJ (2004). Biomechanics of tendon injury and repair. J. Biomech..

[7] Sharma P, Maffulli N (2005). Tendon injury and tendinopathy: Healing and repair. J. Bone Joint Surg. Am..

[8] Mosier SM, Lucas DR, Pomeroy G, Manoli A (1998). Pathology of the posterior tibial tendon in posterior tibial tendon insufficiency. Foot Ankle Int..

[9] Satomi E, Teodoro WR, Parra ER, Fernandes TD, Velosa APP, Capelozzi VL (2008). Changes in histoanatomical distribution of types I, III and V collagen promote adaptative remodeling in posterior tibial tendon rupture. Clinics.

[10] Tresoldi I, Oliva F, Benvenuto M, Fantini M, Masuelli L, Bei R (2013). Tendon’s ultrastructure. Muscles Ligaments Tendons J..

[11] Gonçalves-Neto J, Witzel SS, Teodoro WR, Carvalho-Junior AE, Fernandes TD, Yoshinari HH (2002). Changes in collagen matrix composition in human posterior tibial tendon dysfunction. Joint Bone Spine.

[12] Teodoro WR, Velosa AP, Witzel SS, Garippo AL, Farhat C, Parra ER (2004). Architectural remodeling in lungs of rabbits induced by type V collagen immunization: A preliminary morphologic model to study diffuse connective tissue diseases. Pathol. Res. Pract..

[13] Favata M, Beredjiklian PK, Zgonis MH, Beason DP, Crombleholme TM, Jawad AF (2006). Regenerative properties of fetal sheep tendon are not adversely affected by transplantation into an adult environment. J. Orthop. Res..

[14] Stalling SS, Nicoll SB (2008). Fetal ACL fibroblasts exhibit enhanced cellular properties compared with adults. Clin. Orthop. Relat. Res..

[15] Beredjiklian PK, Favata M, Cartmell JS, Flanagan CL, Crombleholme TM, Soslowsky LJ (2003). Regenerative versus reparative healing in tendon: A study of biomechanical and histological properties in fetal sheep. Ann. Biomed. Eng..

[16] Stanley RL, Fleck RA, Becker DL, Goodship AE, Ralphs JR, Patterson-Kane JC (2007). Gap junction protein expression and cellularity: Comparison of immature and adult equine digital tendons. J. Anat..

[17] Chuen FS, Chuk CY, Ping WY, Nar WW, Kim HL, Ming CK (2004). Immunohistochemical characterization of cells in adult human patellar tendons. J. Histochem. Cytochem..

[18] Hosaka Y, Kirisawa R, Ueda H, Yamaguchi M, Takehana K (2005). Differences in tumor necrosis factor (TNF)alpha and TNF receptor-1-mediated intracellular signaling factors in normal, inflamed and scar-formed horse tendons. J. Vet. Med. Sci..

[19] Russo V, Mauro A, Martelli A, Di Giacinto O, Di Marcantonio L, Nardinocchi D (2015). Cellular and molecular maturation in fetal and adult ovine calcaneal tendons. J. Anat..

[20] Shukunami C, Takimoto A, Nishizaki Y, Yoshimoto Y, Tanaka S, Miura S (2018). Scleraxis is a transcriptional activator that regulates the expression of Tenomodulin, a marker of mature tenocytes and ligamentocytes. Sci. Rep..

[21] Holm-Pedersen P, Viidik A (1972). Tensile properties and morphology of healing wounds in young and old rats. Scand. J. Plast. Reconstr. Surg..

[22] Ippolito E, Natali PG, Postacchini F, Accinni L, De Martino C (1980). Morphological, immunochemical, and biochemical study of rabbit achilles tendon at various ages. J. Bone Joint Surg. Am..

[23] Whitby DJ, Ferguson MW (1991). The extracellular matrix of lip wounds in fetal, neonatal and adult mice. Development.

[24] Moore KL, Persaud TVN, Torchia MG (2008). The Developing Human: Clinically Oriented Embryology.

[25] Parra ER, Aguiar AC, Teodoro WR, de Souza R, Yoshinari NH, Capelozzi VL (2009). Collagen V and vascular injury promote lung architectural changes in systemic sclerosis. Clin. Respir. J..

[26] Gundersen HJ, Bagger P, Bendtsen TF, Evans SM, Korbo L, Marcussen N (1988). The new stereological tools: Dissector, fractionator, nucleator and point sampled intercepts and their use in pathological research and diagnosis. APMIS.

[27] Avilion AA, Nicolis SK, Pevny LH, Perez L, Vivian N, Lovell-Badge R (2003). Multipotent cell lineages in early mouse development depend on SOX2 function. Genes Dev..

[28] Russo V, Berardinelli P, Martelli A, Di Giacinto O, Nardinocchi D, Fantasia D (2006). expression of telomerase reverse transcriptase subunit (TERT) and telomere sizing in pig ovarian follicles. J. Histochem. Cytochem..

[29] Tozer S, Duprez D (2005). Tendon and ligament: Development, repair and disease. Birth Defects Res. C Embryo Today..

[30] Romanic AM, Adachi E, Kadler KE, Hojima Y, Prockop DJ (1991). Copolymerization of pNcollagen III and collagen I. pNcollagen III decreases the rate of incorporation of collagen I into fibrils, the amount of collagen I incorporated, and the diameter of the fibrils formed. J. Biol. Chem..

[31] Hansen KA, Weiss JA, Barton JK (2002). Recruitment of tendon crimp with applied tensile strain. J. Biomech. Eng..

[32] Connizzo BK, Han L, Birk DE, Soslowsky LJ (2016). Collagen V-heterozygous and -null supraspinatus tendons exhibit altered dynamic mechanical behavior at multiple hierarchical scales. Interface Focus..

[33] Zou D, Vigen M, Putnam AJ, Cao C, Tarlé AS, Guinn T, Kaigler D (2022). Phenotypic, trophic, and regenerative properties of mesenchymal stem cells from different osseus tissues. Cell Tissue Res..

[34] Lui PPY (2013). Identity of tendon stem cells–how much do we know?. J. Cell Mol. Med..

[35] Caplan AI (2009). Why are MSCs therapeutic? New data: New insight. J. Pathol..

[36] Goodman SA, May SA, Heinegård D, Smith RKW (2004). Tenocyte response to cyclical strain and transforming growth factor beta depends upon age and site of origin. Biorheology.

